# A comparison of open, laparoscopic, and robotic radical nephrectomy with tumor thrombectomy from the intercontinental collaboration on renal cell carcinoma

**DOI:** 10.1007/s11701-025-02424-z

**Published:** 2025-06-04

**Authors:** Maxwell Sandberg, Gregory Russell, Jacob Malakismail, Mitchell Hayes, Reuben Ben David, Justin Miller, Kartik Patel, Brejjette Aljabi, Seok-Soon Byun, Oscar Rodriguez Faba, Donato Cannoletta, Tatiana Letowski, Gustavo Villoldo, Patricio Garcia Marchinena, Thiago Mourao, Gaetano Ciancio, Charles C. Peyton, Rafael Zanotti, Philippe E. Spiess, Reza Mehrazin, Diego Abreu, Stenio de Cassio Zequi, Alejandro Rodriguez

**Affiliations:** 1 Department of Urology, Atrium Health Wake Forest Baptist Medical Center, Winston Salem, NC USA; 2 Department of Biostatistics, Wake Forest University School of Medicine, Winston Salem, NC USA; 3 Rosalind Franklin University of Medicine and Science, Chicago, IL USA; 4 Department of Genitourinary Oncology, H. Lee Moffitt Cancer Center & Research Institute, Tampa Bay, FL USA; 5 Department of Urology, Icahn School of Medicine at Mount Sinai, New York, NY USA; 6 University of South Florida Health Morsani College of Medicine, Tampa Bay, FL USA; 7 Department of Urology, University of Alabama Birmingham Medical Center, Birmingham, AL USA; 8 Department of Urology, Seoul National University Bundang Medical Center, Seoul, South Korea; 9 Department of Urology, Puigvert Foundation, Barcelona, Spain; 10 Department of Urology, Instituto Alexander Fleming, Buenos Aires, Argentina; 11 Department of Urology, Hospital Italiano, Buenos Aires, Argentina; 12 Department of Urology, A.C. Camargo Cancer Institute, Sao Paulo, Brazil; 13 Department of Urology and Transplant Surgery, University of Miami Miller School of Medicine, Miami, FL USA; 14 Department of Urology, Hospital Pasteur, Montevideo, Uruguay

**Keywords:** Thrombus, Renal cell carcinoma, Robotic, Urology, Laparoscopic

## Abstract

The gold standard treatment for renal cell carcinoma with a tumor thrombus (RCC-TT) is radical nephrectomy with tumor thrombectomy (RN-TT). Operative approaches to this can be done open (ORN-TT), laparoscopic (LRN-TT), or robotic (RRN-TT). The purpose of this study was to compare overall survival (OS), cancer-specific survival (CSS), and metastasis-free survival (MFS) between open, laparoscopic, and robotic approaches to RN-TT using the Intercontinental Collaboration on Renal Cell Carcinoma (ICORCC) database. Patient records were reviewed from the ICORCC database. All patients included in the study underwent RN-TT for RCC-TT from 1999 to present. Tumor thrombus level was graded using the Neves classification system. Statistical analysis was carried out using analysis of variance, chi-squared test, and Kaplan–Meier survival curves with log-rank test to compare outcomes by surgical approach. A total of 392 patients were included. There were 308 ORN-TT, 61 LRN-TT, and 23 RRN-TT cases. On Kaplan–Meier analysis, OS and CSS were not significantly different by approach (*p* > 0.05). MFS was significantly lower in RRN-TT patients (*p* = 0.030). Operative time was the longest in ORN-TT, followed by LRN-TT, and RRN-TT the quickest (*p* = 0.011). Blood transfusion rates were significantly lower in RRN-TT relative to ORN-TT (*p* < 0.001). Rates of lymph node dissection, soft tissue margin positivity, and cytoreductive surgery were alike (*p* > 0.05). There is no definitive superiority of one operative approach compared to another. RRN-TT may result in worse MFS for patients, which calls for further investigation, but this is not certain. Ultimately, the risks, benefits, and resources the surgeon has at his/her disposal should all play in the final operative choice of RN-TT for the patient.

## Introduction

Renal cell carcinoma with tumor thrombus (RCC-TT) is seen in approximately 4–11% of patients diagnosed with renal cell carcinoma (RCC) [1, 2]. The diagnosis carries concerns for both surgeons and patients alike. The significance of TT in RCC is variable with respect to patient survival [3–5]. Nevertheless, it is established that patients with RCC-TT tend to have greater tumor sizes, higher cancer grades and stages, along with a greater propensity for distant metastases [6]. RCC-TT patients are classified by TT level, with a variety of classification systems used [7–9]. TT can be found anywhere from the level of the renal vein all the way up to the right atrium. As TT level increases, incidence tends to go down, with ~ 10–18% of TT in the renal vein, 4–23% in the inferior vena cava, and ~ 1–10% to the level of the right atrium [6, 10]. Mainstay treatment is radical nephrectomy with tumor thrombectomy (RN-TT) by a urologic surgeon which may also involve multidisciplinary collaboration with other surgical teams like cardiothoracic surgery and vascular surgery.

There are a multitude of surgical considerations that must be noted by the surgical team prior to RN-TT. These have been reported extensively. Highlights include adequate preoperative imaging to fully assess the level of the TT, preoperative anticoagulation when appropriate, complete resection of both the tumor and TT, appropriate control of bleeding intraoperatively, which can include cardiopulmonary bypass at times, and adequate postoperative care [11–13]. When adequate surgical control is obtained, both overall survival (OS) and cancer-specific survival (CSS) benefits have been observed [14, 15]. What is not as well studied is the impact that the surgical approach has on RCC-TT, namely perioperative outcomes, metastasis-free survival (MFS), CSS, and OS.

The three approaches to RN-TT are open RN-TT (ORN-TT), laparoscopic RN-TT (LRN-TT), and robotic RN-TT (RRN-TT). Traditionally, ORN-TT was employed for surgical resection, however, in 2002 the first-ever laparoscopic approach was reported for RN-TT and in 2015 LRN-TT was described for a level IV thrombus at the atrium of the heart [16, 17]. With the advent of the da Vinci robotic platform by Intuitive Surgical Systems™ into urology, RRN-TT was first reported in 2011 for level I and II TT and has now been utilized for all TT levels [18–20]. Despite extensive reporting on surgical technique, there is a paucity of research that compares ORN-TT, LRN-TT, and RRN-TT. The purpose of this study was to compare ORN-TT, LRN-TT, and RRN-TT with a specific focus on MFS, CSS, and OS.

## Methods

This was a retrospective multi-institutional analysis under IRB00096722 of patients diagnosed with RCC-TT who subsequently underwent RN-TT for treatment of their disease via an open, laparoscopic, or robotic approach. All patients came from the Intercontinental Collaboration on Renal Cell Carcinoma (ICORCC) database. ICORCC is a multi-institutional and multi-continental group that specifically focuses on research around RCC-TT. Cases in this analysis came from the United States of America, Mexico, Peru, Uruguay, Bolivia, Chile, Argentina, Brazil, Spain, and South Korea. Patients were divided into three groups based on operative choice between ORN-TT, LRN-TT, RRN-TT. TT level was categorized using the Neves classification system [7]. Surgical methodology was not standardized in the cohort, but all level IV RN-TT utilized cardiopulmonary bypass. ORN-TT was performed via either subcostal, thoracoabdominal or a modified Gibson incision. LRN-TT was either pure laparoscopic or hand-assist, and no differentiation was made for the purposes of this study. For level IV LRN-TT, surgery was performed in a manner like Shao et al. [17]. RRN-TT was done with either the da Vinci Si™ or Xi™ platform. Port placement varied based on operating surgeon preference, but was most often similar to previously described techniques [21, 22]. For left-sided renal tumors, clamping of the right renal artery and vein was often but not always employed by the operating surgeon, according to published methodology [22]. The order of clamping/vascular block also varied by surgeon and/or institution as did the method used to perform this and order of unclamping as previously described [23].

A variety of pre-, peri-, and postoperative variables were collected on patients in the study. Preoperative tumor size was determined by measuring the largest tumor dimension of preoperative imaging, most often computerized tomography (CT) or magnetic resonance imaging (MRI). Cytoreductive nephrectomy was defined as RN-TT performed on patients who were confirmed to be metastatic by imaging prior to surgery. Intraoperative blood loss and intraoperative/postoperative transfusion information were available for ORN-TT and RRN-TT patients only. Tumors were graded according to the International Society of Urological Pathology grading system and staged according to the TNM Classification system [24, 25]. Neoadjuvant systemic therapy and systemic therapy administration after RN-TT were recorded. Cancer-specific death was determined by a pathologist and/or oncologist. Metastasis were determined by either CT or MRI and the date of first imaging showing metastasis was used to calculate MFS.

Statistical analysis was done to compare outcomes between operative approaches. We based our approach for analysis on the Central Limit Theorem, following that the distribution of sample means will tend to be normal, even if the population distribution is not normal, so long as the sample size is sufficiently large. When comparing groups, generally there is no need to worry about normality in the population itself. With respect to multiple comparisons, we viewed this set of analyses as exploratory and did not adjust the p-value; each test was performed at a pre-specified level of significance of 0.05 for consistency across all tests. Analysis of variance (ANOVA) was used to compare means of continuous variables and chi-squared test was used for categorical variables. For mean intraoperative blood loss, an independent samples t-test was used as a comparison was done for only two groups (ORN-TT and RRN-TT). Kaplan–Meier survival analysis with log-rank test was done to compare MFS, CSS, and OS between ORN-TT, LRN-TT, and RRN-TT. All statistics were run with SPSS Statistics Version 28 (Armonk, NY) or SAS Statistics Version 9.4 (SAS Institute Inc.).

## Results

The full complement of continuous variable data in the population is found in Table 1 and categorical variable data in Table 2. A total of 392 patients were included (121 female and 271 male). Of all operations, 23 were RRN-TT, 61 LRN-TT, and 308 ORN-TT. No difference in operative choice existed by gender (*p* > 0.05). Charlson comorbidity index, Karnofsky performance status, prevalence of diabetes, prevalence of chronic kidney disease, prevalence of coronary artery disease, prevalence of hypertension, prevalence of hyperlipidemia, prevalence of active smokers, body mass index, and age were similar by approach (*p *> 0.05). Race differed by approach (*p* < 0.001). White patients made up most subjects undergoing ORN-TT (73%) and RRN-TT (56.5%) and Hispanic/Latino patients made up the most subjects undergoing LRN-TT (81%). Preoperative tumor size was similar (*p* > 0.05). Use of neoadjuvant systemic therapy did not differ by approach (*p* > 0.05). Seventy-eight patients (25.3%) underwent cytoreductive nephrectomy in the ORN-TT group, 12 (52.2%) in the LRN-TT group, and six (26.1%) in the RRN-TT group. Operative time (OT) was longest in open surgery, followed by robotic, and laparoscopic was the quickest (*p* = 0.011). Length of stay was similar (*p* > 0.05) between ORN-TT, LRN-TT, and RRN-TT. TT level distribution using the Neves classification system differed by approach (*p* = 0.041). RRN-TT had the smallest percentage of level IV TT (4.3%) and the greatest percentage of level I TT (47.8%). ORN-TT had a significantly greater proportion of left-sided tumors (38.3%; *p* < 0.001). Rates of lymph node dissection, mean lymph node yield, positive lymph nodes, negative lymph nodes, soft tissue margin positivity, cytoreductive surgery, development of postoperative metastasis, and initial number of metastatic sites were alike (*p* > 0.05). For patients undergoing cytoreductive nephrectomy, there was an equal distribution in the location of metastatic disease by approach (*p* > 0.05). Tumor stage was different, and open patients had a significantly greater proportion of T3b (56.9%) and T3c (15.6%) disease and robotic subjects had a significantly greater proportion of T3a disease (47.8%; *p* = 0.003). Renal vein margin positivity was greatest in RRN-TT (52.2%; *p* = 0.005). Tumor grade, tumor subtype, and prevalence of sarcomatoid variants were similar between all three approaches (*p* > 0.05). Tumor necrosis was more prevalent in ORN-TT (66.7%) and RRN-TT (56.5%) patients relative to LRN-TT (44.2%; *p* = 0.010). Mean intraoperative blood loss did not differ between ORN-TT and RRN-TT, but the rate of blood transfusion was significantly higher in ORN-TT cases (57.7%; *p* < 0.001). All but three (93.3%) ORN-TT level IV cases required an intraoperative blood transfusion, and the level IV RRN-TT case did not. Rates of systemic therapy after RN-TT were similar by approach (*p* > 0.05).

**Table 1:** Continuous variables in the study population

Variable	ORNTT	LRNTT	RRNTT	Total mean	*P*-value
Age at operation (years)	62.3 (11.3)	62.3 (10.6)	63 (14.8)	62.3 (11.4)	0.961
Body mass index	28 (6)	28.5 (5.1)	27.7 (5.6)	28 (5.8)	0.759
Charlson comorbidity index	4.2 (3.4)	4 (1.6)	5.6 (2.5)	4.3 (3.3)	0.426
Karnofsky	85.5 (10.2)	83.6 (11.4)	91.4 (3.8)	85.5 (10.2)	0.213
Preoperative tumor size	9.8 (3.8)	8.8 (3.7)	8.9 (3.3)	9.6 (3.8)	0.142
Operative time (minutes)	306.5 (142.4)	255.4 (92.5)	259.7 (78.7)	293.9 (132.5)	0.011
Length of stay (days)	12.1 (58.4)	6.7 (6.3)	6.9 (11.4)	11 (51.7)	0.712
Postoperative tumor size	9.7 (3.6)	8.9 (3.6)	9.4 (3.3)	9.5 (3.6)	0.363
Lymph node yield	3.6 (4.3)	3.1 (2.5)	7.3 (8.4)	3.8 (4.5)	0.099
Positive lymph nodes	0.5 (1.3)	0.6 (1.2)	0.4 (0.5)	0.5 (1.2)	0.89
Negative lymph nodes	3.4 (4.2)	2.7 (4.6)	5.7 (8.0)	3.4 (4.5)	0.251
Initial number of metastatic sites	1.6 (0.8)	1.9 (1.2)	2.1 (2.5)	1.7 (1.1)	0.124
Blood loss (mL)	1485.2 (2500.4)	–	240.4 (217.8)	1432.9 (2460.2)	0.074
Follow-up (years)	3.2 (4.2)	3.5 (5.5)	1.7 (1.9)	3.2 (4.3)	0.222

The following table shows interval variables in the study population. Each operative approach is shown with associated *p*-values for comparison. Numbers are means with standard deviation in parentheses

**Table 2:** Categorical variable comparisons in the study population

Variable	ORNTT	LRNTT	RRNTT	Total	*P*-value
Total N	308	61	23	392	–
Race					
White	208/285 (73)	11/60 (18.3)	13 (56.5)	232	< 0.001
Black	25/285 (8.8)	2/60 (3.3)	0 (0)	27	
Hispanic/Latino	44/285 (15.4)	47/60 (78)	9 (39.1)	100	
Asian	8/285 (2.8)	0/60 (0)	1 (4.3)	9	
Female	96 (31.2)	17 (27.9)	8 (34.7)	121	0.804
Diabetes Mellitus	52/208 (25)	20 (32.8)	6 (26)	78	0.48
Chronic kidney disease	29/208 (14)	10 (16.4)	4 (17.4)	43	0.832
Hypertension	212 (68.8)	47 (77)	17 (73.9)	276	0.927
Hyperlipidemia	51 (16.6)	8 (13.1)	6 (26.1)	65	0.149
Coronary artery disease	28/274 (10.2)	8 (13.1)	2 (8.7)	30	0.363
Active smoker	28/199 (14.1)	13 (21.3)	5 (21.7)	16	0.351
Renal vein level					
I	86/282 (30.5)	28/60 (46.7)	11 (47.8)	125	0.041
II	66/282 (23.4)	17/60 (28.3)	4 (17.4)	87	
III	85/282 (30.1)	9/60 (15)	7 (30.4)	101	
IV	45/282 (16)	6/60 (10)	1 (4.3)	52	
Side					
Right	82/133 (61.7)	43/48 (89.6)	15/19 (79)	140	< 0.001
Left	51/133 (38.3)	5/48 (10.4)	4/19 (21.1)	60	
Stage					
T1b	0/299	1/58 (1.7)	0/22 (0)		
T3a	59/299 (19.7)	23/58 (39.7)	11/22 (50)	93	0.003
T3b	170/299 (56.9)	26/58 (44.8)	8/22 (36.3)	204	
T3c	46/299 (15.4)	6/58 (10.3)	2/22 (9.1)	54	
T4	24/299 (8)	3/58 (5.2)	1/22 (4.50)	28	
Grade					
0	1/293 (0.3)	0/56 (0)	1/22 (4.5)	2	0.129
1	6/293 (2)	1/56 (1.8)	0/22 (0)	7	
2	46/293 (15.7)	10/56 (17.9)	3/22 (13.6)	59	
3	138/293 (47.1)	18/56 (32.1)	9/22 (41)	165	
4	102/293 (34.8)	27/56 (48.2)	9/22 (41)	138	
Subtype					
Clear cell	256/300 (85.3)	48/60 (80)	18 (78.3)	322	0.662
Papillary	23/300 (7.7)	5/60 (8.3)	2 (8.7)	30	
Other	21/300 (7)	7/60 (11.7)	3 (13)	31	
Sarcomatoid	34/206 (16.5)	6/60 (10)	3 (13)	43	0.445
Necrosis	130/195 (66.7)	27 (44.2)	13 (56.5)	170	0.010
Lymph node dissection	118/282 (41.8)	18/53 (34)	9/21 (42.9)	145	0.552
Soft tissue margin positive	59/204 (23)	4/24 (16.7)	1/13 (7.7)	64	0.125
Renal vein margin positive	100/191 (52.4)	41/53 (77.4)	12/21 (57.1)	153	0.005
Blood transfusion	169/293 (57.7)	-	0/13	169	< 0.001
Preoperative metastasis	78 (25.3)	12 (19.7)	6 (26.1)	105	0.825
Preoperative metastatic location					
Lung	34		6	52	0.619
Bone	10	3	2	15	0.939
Brain	3	0	0	3	0.457
Retroperitoneum	17	8	1	26	0.179
Adrenal	7	2	0	9	0.513
Para-aortic nodes	1	0	0	1	0.598
Liver	11	5	0	16	0.214
Other	8	0	2	10	0.423
Neoadjuvant systemic therapy	16/211 (7.6)	4 (6.6)	0	20	0.388
Postoperative systemic therapy	115/171 (39.8)	18 (31.6)	13/22 (59.1)	99	0.081
Metastasis postoperative	58 (18.8)	14 (23.0)	7 (30.4)	79	0.934
Dead	133 (43.2)	25 (41.0)	11 (47.8)	169	0.851
Cancer-specific death	97/133 (72.9)	13/25 (52)	9/11 (81.8)	119	0.001

The following table compares categorical variables between each operative approach in the study. Associated p-values are provided. Numbers are totals with percentage of the cohort in parentheses

**Fig. 1:**
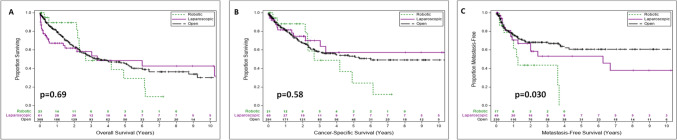
Kaplan–Meier overall survival analysis. The following figure compares **A**-overall survival, **B**-cancer-specific survival, and **C**-metastasis-free survival by operative approach. Proportion of patients surviving at each time interval is shown on the y-axis and time is in years and shown on the x-axis. Number at risk tables are shown below each plot. Log-rank *p*-value is provided

Mean follow-up in the entire cohort was 3.2 years. For ORN-TT patients it was 3.2 years, LRN-TT patients 3.5 years, and RRN-TT patients 1.7 years, which did not significantly differ (*p* > 0.05). Survival analysis data is shown in Table 3. Median overall survival in the ORN-TT group was 3.5 years, 3.3 years in the LRN-TT group, and 2.7 years in the RRN-TT group. ORN-TT CSS was 6 years and 2.7 years in RRN-TT patients. Median CSS was not reached in the LRN-TT grouping, but there was 57% survival at 3.3 years, with no cancer-specific deaths afterwards. Metastasis-free survival was 14.5 years in the ORN-TT group, 6.2 years in the LRN-TT group, and 1.3 years in the RRN-TT group. Figure 1 represents Kaplan–Meier survival analysis in the patient population. On Kaplan–Meier analysis, OS and CSS were not significantly different by approach (*p* > 0.05). MFS was significantly lowest in the RRN-TT group (*p* = 0.030).

**Table 3:** Survival analysis

Survival	ORN-TT	LRN-TT	RRN-TT	*P*-value
Overall survival (years)	3.5; *n* = 308	3.3; n = 61	2.7; *n* = 23	0.69
Cancer-specific survival (years)	6.0; *n* = 291	Not reached; n = 60	2.7; *n* = 21	0.58
Metastasis-free survival (years)	14.5; *n* = 230	6.2; *n* = 49	1.3; *n* = 17	0.030

The following table provides median survival data on each operative approach and total number of patients in each cohort for each survival analysis. Log-rank *p*-values are also provided

## Discussion

This study is one of the largest comparisons of ORN-TT, LRN-TT, and RRN-TT in the world to date. We draw from a diverse set of patients across the globe strengthening the external validity of the results. Prior research has focused on comparing operative approaches to RN-TT, showing no difference in OS, CSS, and MFS [26]. Likewise, we also saw no difference in OS and CSS but did identify a shorter MFS with RRN-TT. Further, multiple non-survival outcomes differed by operative approach. Multiple studies have compared operative outcomes by approach but to date no randomized control trials exist, and sample size is often an issue in the data that has been published prior.

OS was not affected by the operative approach in our study. Likewise, CSS did not appear to change between ORN-TT, LRN-TT, and RRNTT patients. In a propensity-matched cohort study, Zhang et al. compared 88 ORN-TT, 88 LRN-TT, and 22 RRN-TT and saw no difference in OS nor CSS [26]. Rose et al. compared 27 ORN-TT patients to 24 RRN-TT, also noting no difference in OS. Gu et al. compared level I and II RCC-TT patients who either underwent ORN-TT (37 patients) and RRN-TT (31 patients) [27]. Like others, the authors also saw no difference in OS or CSS. Mean intraoperative blood loss was not significantly different in our analysis between ORN-TT and RRN-TT patients but the rate of blood transfusion (intraoperative or postoperatively) in the robotic cohort was significantly lower, notably at 0%. Zhang et al. saw that RRN-TT had the shortest OT, least blood loss, and fewest transfusions after RN-TT [26]. Rose et al. also concluded that RRN-TT had lower blood loss and blood transfusions compared to ORN-TT, along with a shorter LOS [27]. Vuong et al. compared 30 ORN-TT and 10 RRN-TT level I and II RN-TT cases, concluding that RRNTT had lower blood loss and LOS but longer OT [28]. We recognize our results may in part be due to the discrepancy in TT level between open and robotic cases, as greater TT levels have been associated with more blood loss/transfusion requirements [29, 30]. Despite this, our results are in line with much of the established literature that RRN-TT, a more minimally invasive approach to RN-TT, may lessen transfusion requirements. No LOS difference was identified in our study, but OT was significantly longer in ORN-TT relative to LRN-TT and RRN-TT. Our survival findings are relevant given that a more minimally invasive approach does not appear to compromise OS nor CSS. Additionally, it may afford the patient a shorter time on the operating table with a lower risk of requiring a blood transfusion.

Metastatic RCC is well-established to be associated with shorter OS and CSS [31]. Current study indicates that MFS is unlikely to be different between ORN-TT, LRN-TT, and RRN-TT. Zhang et al. saw no difference in MFS during their propensity-matched study [26]. Rose et al. did not notice a difference in recurrence-free survival rates [27]. Gu et al. also saw no difference in progression-free survival [32]. Our study did identify a significantly shorter MFS time in RRN-TT relative to ORN-TT and LRN-TT, which is arguably the most important result from our analysis. This is key as our findings indicate that a more minimally invasive approach to RN-TT could compromise recurrence but must be viewed in the context of multiple limitations. While statistical significance was not identified in follow-up times by operative approach, the lowest mean follow-up time was noted in the RRN-TT group. It is possible that a shorter follow-up time in the robotic cohort accounts for worse MFS. Additionally, patient selection bias for those who underwent robotic surgery could play a role in these results, especially given the retrospective nature of our study. Prior research has highlighted the importance of adequate patient selection when performing RN robotically [33]. Perioperative management factors may also play a role here, as no RRN-TT patients received neoadjuvant systemic therapy. In the past, we have published that neoadjuvant systemic therapy may affect survival parameters in RN-TT [34]. Others have also noted the importance of anatomical diagramming and surgeon experience when performing robotic removal of a TT, which varied significantly in the cohort and could be a reason for the lower MFS in the RRN-TT grouping [35]. Likewise, the variability in surgical technique across institutions enrolled in the study may account for the difference in MFS observed. As an example, the order of vascular block and technique varied by surgeon. Similar to reports by Luo et al., some surgeons elected to first block the caudal IVC, followed by the contralateral renal vein, and then cephalad IVC, whereas others elected a different order or did not block all three [23]. Patient tumor pathology also was not equivalent, as a greater portion of ORN-TT and RRN-TT patients had necrosis relative to the LRN-TT group. Necrosis has been associated with worse progression-free survival in RCC [36]. Ultimately, the worse MFS seen in the RRN-TT group is not definitive but raises questions about a robotic approach to RN-TT that requires more investigation.

An important observation we saw in the cohort was a significant difference in tumor laterality by operative approach. Only 10.4% of LRN-TT and 21% of RRNTT patients had a left sided tumor. By contrast, 38.3% of patients in the ORN-TT had a left-sided tumor, which was significantly different from the other approaches. This observation was also noted by Zhang et al. in their propensity-matched analysis of ORN-TT, LRN-TT, and RRN-TT [26]. The authors noted left-sided RN-TT cases had greater blood loss and postoperative complications [26]. Left-sided RN-TT is generally thought to be more challenging to perform, often due to the proximity of the left renal vein to the superior mesenteric artery, repositioning requirements intraoperatively, and a longer renal vein which can complicate hemostasis [22, 26, 37]. Although appropriate techniques have been described to successfully perform RN-TT via LRN-TT or RRN-TT, this study indicates that urologists still may prefer the traditional open approach when dealing with a left-sided renal tumor with TT.

There are multiple limitations we acknowledge in this analysis. First, this study is subject to the inherent biases of any retrospective review, namely selection bias in the cohort. Second, we did not have complete data on every patient included in the analysis. Further, there was not an equal distribution of ORN-TT, LRN-TT, and RRN-TT patients. While the total cohort we report on is substantial, the subgroup size for RRN-TT is fairly limited in size, which may hinder the statistical power for comparison and generalizability of our findings. The multi-institutional nature of this paper is a strength, however, surgical technique was not standardized, which we have discussed particularly in relation to the difference in MFS and is another limitation. Moreover, we did not report information on conversion from LRN-TT or RRN-TT procedures to ORN-TT intraoperatively or postoperative complications. While we do describe estimated blood loss and transfusion requirements, the data is incomplete, and no LRN-TT information was available to report. Despite these weaknesses, this study has strengths like its diverse patient population from four different continents, a significantly larger sample size compared to similar publications, and excellent follow-up data.

## Conclusions

RN-TT can be performed open, laparoscopic, or robotic. Each operative approach has benefits and drawbacks associated with it. OS and CSS do not appear to be affected by surgical choice, but MFS may be worse with a robotic approach, although further study is needed, and transfusion risk may be lessened with an RRN-TT approach. Given this information, more minimally invasive techniques should be considered by urologists when making surgical approach selection. Concurrently, it should be recognized that many countries lack sufficient access to surgical robots, and an open approach does not sacrifice OS, CSS, or MFS. This retrospective analysis is one of the largest to ever be reported. Our findings call for prospective analysis for confirmation.

## Data Availability

Data that was used in the manuscript is not publicly available due to patient protection, but is available in de-identified form upon reasonable request to the corresponding author.
