# Correction: A comparison of open, laparoscopic, and robotic radical nephrectomy with tumor thrombectomy from the intercontinental collaboration on renal cell carcinoma

**DOI:** 10.1007/s11701-025-02492-1

**Published:** 2025-06-23

**Authors:** Maxwell Sandberg, Gregory Russell, Jacob Malakismail, Mitchell Hayes, Reuben Ben David, Justin Miller, Kartik Patel, Brejjette Aljabi, Seok-Soon Byun, Oscar Rodriguez Faba, Donato Cannoletta, Tatiana Letowski, Gustavo Villoldo, Patricio Garcia Marchinena, Thiago Mourao, Gaetano Ciancio, Charles C. Peyton, Rafael Zanotti, Philippe E. Spiess, Reza Mehrazin, Diego Abreu, Stenio de Cassio Zequi, Alejandro Rodriguez

**Affiliations:** 1 Department of Urology, Atrium Health Wake Forest Baptist Medical Center, Winston Salem, NC USA; 2 Department of Biostatistics, Wake Forest University School of Medicine, Winston Salem, NC USA; 3 Rosalind Franklin University of Medicine and Science, Chicago, IL USA; 4 Department of Genitourinary Oncology, H. Lee Moffitt Cancer Center & Research Institute, Tampa Bay, FL USA; 5 Department of Urology, Icahn School of Medicine at Mount Sinai, New York, NY USA; 6 University of South Florida Health Morsani College of Medicine, Tampa Bay, FL USA; 7 Department of Urology, University of Alabama Birmingham Medical Center, Birmingham, AL USA; 8 Department of Urology, Seoul National University Bundang Medical Center, Seoul, South Korea; 9 Department of Urology, Puigvert Foundation, Barcelona, Spain; 10 Department of Urology, Instituto Alexander Fleming, Buenos Aires, Argentina; 11 Department of Urology, Hospital Italiano, Buenos Aires, Argentina; 12 Department of Urology, A.C. Camargo Cancer Institute, Sao Paulo, Brazil; 13 Department of Urology and Transplant Surgery, University of Miami Miller School of Medicine, Miami, FL USA; 14 Department of Urology, Hospital Pasteur, Montevideo, Uruguay

**Correction: Journal of Robotic Surgery (2025) 19:269** 10.1007/s11701-025-02424-z

In the original version of this article, the affiliation details for the author Donato Cannoletta were incorrectly given as ‘Department of Urology, Instituto Alexander Fleming, Buenos Aires, Argentina’ but should have been ‘Department of Urology, Puigvert Foundation, Barcelona, Spain’.

